# Salicylate of Ammonium as a Substitute for Salicylic Acid

**Published:** 1876-12

**Authors:** 


Salicylate of Ammonium as a Substitute for Salicylic Acid for Internal Use.—J. F. Martenson (*Centralbl. f. Med.*, No. 19, 1876, p. 352) suggests the use of this salt, which is easily made by saturating the aqueous solution of the acid with ammonia or its carbonate. It is easily soluble in water and alcohol, has a sweetish taste, and is tolerably stable. One hundred parts contain about eighty-nine parts of salicylic acid.

